# Association of the TLR4 Asp299Gly polymorphism with lung function in relation to body mass index

**DOI:** 10.1186/1471-2466-9-46

**Published:** 2009-09-21

**Authors:** Punam Pahwa, Chandima P Karunanayake, Donna C Rennie, Yue Chen, David A Schwartz, James A Dosman

**Affiliations:** 1 Canadian Centre for Health and Safety in Agriculture; 103, Hospital Drive, P.O. Box 120, R.U.H., Saskatoon, Saskatchewan, S7N 0W8, Canada; 2 Department of Community Health and Epidemiology; Health Science Building, 107, Wiggins Road, University of Saskatchewan, Saskatoon, Saskatchewan, S7N 5E5, Canada; 3 College of Nursing, 107, Wiggins Road, University of Saskatchewan, Saskatoon, Saskatchewan, S7N 5E5, Canada; 4 Department of Epidemiology and Community Medicine, University of Ottawa, 451, Smyth Road, Ottawa, Ontario, K1H 8M5, Canada; 5 Division of Pulmonary and Critical Care Medicine, National Jewish Medical and Research Center, 1400, Jackson Street, Denver, Colorado, 80206, USA

## Abstract

**Background:**

Previous studies have shown conflicting results for the association between TLR4 polymorphism (Asp299Gly) and lung function. We investigated the influence of TLR4 Asp299Gly, a polymorphism, on lung function in a community population.

**Methods:**

In 2003, a cross-sectional survey was conducted to assess the respiratory health of residents living in and around the town of Humboldt, Saskatchewan, Canada. There were 2090 adults age 18-79 years who completed a questionnaire that included a medical and smoking history, as well as socio-economic and lifestyle variables. Genetic information and lung function test measurements were available on 1725 subjects (754 males and 971 females) of the 2090 respondents. These subjects were selected for further analysis to investigate the association between TLR4 Asp299Gly genotype and forced expiratory volume in the first second in liters (FEV_1_), forced vital capacity in liters (FVC), FEV_1_/FVC ratio, and forced expiratory flow rate in liters/second (FEF_25-75_). Multivariable linear regression analysis was used to investigate associations.

**Results:**

**A**djusted mean values of FEV_1 _and FVC were significantly different between TLR4 wild type and TLR4 variant groups [Mean ± S.E.: (TLR4 wild type - FEV_1_: 3.18 ± 0.02, FVC: 3.95 ± 0.03; TLR4 variant - FEV_1_: 3.31 ± 0.06, FVC: 4.14 ± 0.07)]. Based on multivariable regression analysis, we observed that body mass index (BMI) was associated with decreased FEV_1_/FVC ratio and FEF_25-75 _in TLR4 variant group but not in wild type group.

**Conclusion:**

BMI may modify the associations of TLR4 Asp299Gly polymorphism with FEV_1_/FVC ratio and FEF_25-75_.

## Background

Toll-like receptor (TLR) proteins have been shown to play an important role in both innate and adaptive immune response in higher vertebrates and have been involved in a number of lung-associated immune responses and pathologies [1-7]. The role of TLR proteins in lung-associated conditions such as airway hyperactivity, allergic asthma, tuberculosis and exposure to endotoxin has been studied [6,7]. TLR4 has been shown to play a role in allergic asthma, perhaps by modulating the Th1 vs. Th2 responses [8]. TLR4 polymorphisms Asp299Gly and Thr399Ile have been associated with the development of respiratory diseases [5]. However, LeVan et al. [9] reported no association between TLR4 Asp299Gly and lung function or wheeze. The prevalence of TLR4 Asp299Gly polymorphism has been demonstrated to be approximately 10-12% in populations dominated by Caucasians [(Rennie DC, Chen Y, Pahwa P, Burch LH, Bharadwaj L, Grover VK, Schwartz DA, Dosman JA: Gender-related associations between toll-like 4 receptor 299 polymorphism and respiratory outcomes, submitted), [3]].

It has been shown that the TLR4 gene may have a major influence on adiposity [10], and metabolic syndrome [11] in addition to its well known role in the immune response. An association between body mass index and reduced lung function has been demonstrated [12,13]. A few studies have shown an association between waist circumference, a component of the metabolic syndrome, adiposity and impaired lung function [14-17]. It has been suggested that waist circumference is a better indicator of metabolic syndrome compared to percentage fat [17]. The association between TLR4 Asp299Gly polymorphism and lung function in relation to obesity (in terms of BMI or waist circumference or other metabolic syndrome components) is an uncharted area. The aim of this paper was to investigate a possible association between the Asp299Gly polymorphism of the TLR4 and the lung function measurements (FEV_1_, FVC, FEF_25_75_, and FEV_1_/FVC ratio) in the presence of potential effect modifiers and/or confounders.

## Methods

### Recruitment

The cross-sectional community study of adults was conducted in 2003 in Humboldt, Saskatchewan, Canada through a household canvass of town residents and through a mail out to rural residents in the surrounding municipality. All participants brought completed questionnaires to a health screening centre located in the town. The study protocol has been previously described [18]. Briefly, 2090 adults in the age group 19-79 years completed a questionnaire that included medical history, smoking history, socio-economic and life style variables. Subjects signed a witnessed informed consent. Prior to the study, approval by the Biomedical Research Ethics Board of the University of Saskatchewan was obtained. Genetic information and lung function test measurements were available on 1725 subjects (754 males and 971 females).

### Questionnaire

The respiratory questionnaire collected information on demographics, medical history, personal smoking history and household smoking. Information on exposure to grain dust was identified by history of grain farming or handling grains.

### Pulmonary function testing

Pulmonary function test variables forced vital capacity (FVC [L]), forced expired volume in one second (FEV_1 _[L]), forced expiratory flow at 25%-75% vital capacity (FEF_25-75 _[L/sec]), and ratio of FEV_1 _to FVC (FEV_1_/FVC [%]) were conducted using *MedGraphics CPF-S system*, (*Medical Graphics Corp. St. Paul, MN 55127, 1992*), according to American Thoracic Society criteria[19]. Percent expected values for pulmonary function test variables were derived from the equations of Crapo et al.[20]

### DNA isolation and genotyping

Blood samples were collected in Qiagen *PAXgene *tubes. Blood cards were spotted for each sample (*S&S, catalog #10538414*) [21]. DNA was isolated on a Gentra Autopure robot (*Qiagen Corporation, Hilden, Germany*), quantified by UV spectrophotometry, and stored at -80°C. Working dilutions of DNA at a concentration of 10 ng/ul were prepared for genotyping. TaqMan allelic discrimination assays for human TLR4 Asp299Gly polymorphism was designed using Primer Express software *(Applied Biosystems, Foster City CA*). Fluorescent probes were ordered with minor groove binding (MGB) protein and non-fluorescent quencher (NFQ). Plasmids carrying mutant (299) and wild type sequences were diluted to a concentration of 20 fg/ul as controls. Primer and probe concentrations were optimized using control plasmids as templates. TaqMan genotype was determined by post-amplification plate reading on an ABI Prism 7900 Sequence Detection System (*Applied Biosystems*). Assays were not available on demand at that time, therefore they were custom designed. The assay was titrated for optimal performance, so the concentration information is important. Sequence and concentration information for HTLR4 299 TqaMan Assays is as follows:

Oligonucleotide   Sequence   Conc. (nM)

D299G wildtype   TET-CCTCGATGATATTATT-MGBNFQ   900

D299G mutant   FAM-CCTCGATGGTATTAT-MGBNFQ   50

299 forward primer   GAAGAATTCCGATTAGCATACTTAGACTACT   200

299 reverse primer   TAATTCTAAATGTTGCCATCCGAA   200

DNA samples that failed to genotype in two assays were submitted for sequencing by fluorescent dye termination sequencing.

### Data Analysis

Descriptive statistics were expressed as mean ± S.E. Comparisons of demographic characteristics between any two groups were conducted by using two-tailed Student's t-test or Pearson's Chi-square test. Multiple linear regression analysis was conducted to determine associations between TLR4 Asp299Gly polymorphism and lung function measurements after adjusting for age, height, sex and smoking. Statistical analyses were performed using SPSS (SPSS Inc, Chicago, IL). In order to investigate the association between TLR4 Asp299Gly polymorphism and lung function, adjusting for other potentially important covariates, the univariate analysis was conducted to determine the candidate variables for the multivariable model [22]. Variables with p-value < 0.20 were selected for the multivariable linear analysis.

## Results

For TLR4 299, 86.4%, were Asp299Asp, 13.4% were Asp299Gly, and 0.2% were Gly299Gly [Rennie DC, Chen Y, Pahwa P, Burch LH, Bharadwaj L, Grover VK, Schwartz DA, Dosman JA: Gender-related associations between toll-like 4 receptor 299 polymorphism and respiratory outcomes, submitted]. The allelic frequencies for the TLR4 Asp299Gly polymorphism were Asp = 93.0% and Gly = 7.0%. The Hardy-Weinberg test for equilibrium was met (p > 0.05).

Demographic characteristics of the study population stratified by sex are shown in Table 1. As demonstrated, there were no differences in mean values for age between males and females. Compared to males there were more females in the BMI category <25 kg/m^2^. The proportion of ever smokers was significantly higher in males (52.4%) compared to females (40.5%). TLR4 Asp299Gly frequency was 15.8% for males and 11.9% for females. Significantly more males (59.5%) were exposed to grain dust compared to females (23.1%).

**Table 1:** Demographic characteristics and TLR4 Asp299Gly stratified by Sex

	**Sex**	
	
	**Male (n = 754)**	**Female (n = 971)**
	**Mean ± SE**	**Mean ± SE**
Age, yrs	51.7 ± 0.55	51.1 ± 0.51
Height, cm	175.5 ± 0.57	162.4 ± 0.51
Weight, Kg	89.8 ± 0.24	74.3 ± 0.20
		
	n(%)	n(%)
Age groups		
< 40 years	180 (23.9%)	246 (25.3%)
40-59 years	320 (42.4%)	393 (40.5%)
≥ 60 years	254 (33.7%)	332 (34.2%)
		
Body Mass Index (Kg/m^2^)		
< 25	132 (17.5%)	289 (32.7%)*
25-30	343 (45.5%)	301 (34.1%)
>30	279 (37.0%)	293 (33.2%)
		
Smoking Status		
Never Smoker	359(47.6%)	578 (59.5%)*
Ever Smoker	395 (52.4%)	393 (40.5%)
		
TLR4299		
Wild-type	635 (84.2%)	855 (88.1%)^†^
Polymorphic	119 (15.8%)	116 (11.9%)
		
Grain Dust		
Not Exposed	305 (40.5%)	747 (76.9%)*
Exposed	449 (59.5%)	224 (23.1%)
		
Household Smoking		
Not Exposed	677 (89.8%)	846 (87.1%)
Exposed	77 (10.2%)	125 (12.9%)

* p < 0.001; ^† ^p < 0.05

Mean values for the pulmonary function test variables FVC (L), FEV_1_(L), FEF_25-75 _(L/sec), and FEV_1_/FVC ratio are shown in Table 2. These results are stratified by (i) sex (male vs. female); (ii) smoking status (never smoker vs. ever smoker) and (iii) TLR4299 genotype (wild type vs. polymorphic). There was no difference in the observed FEV_1 _and FVC values between never and ever smokers. However, ever smokers had significantly lower predicted FEV_1 _and FVC values compared to never smokers. Ever smokers had significantly lower values for FEV_1_/FVC ratio and FEF_25-75 _(both observed and predicted). Compared to the wild type group, those with the polymorphic group had significantly higher observed FEV_1 _and FVC. For other observed or predicted lung function values, no differences were observed between the wild type and polymorphic groups.

**Table 2:** Observed mean value ± standard error and mean values of the % predicted ± standard error for the pulmonary function test variables for sex, smoking status and TLR4 Asp299Gly

	**Sex**		**Smoking Status^#^**		**TLR4 299^#^**	
	
	**Male (n = 754)**	**Female (n = 971)**	**Never Smoker** **(n = 937)**	**Ever Smoker** **(n = 788)**	**Wild type (n = 1490)**	**Polymorphic** **(n = 235)**
**Observed FEV**_1_**(L)**	3.78 ± 0.03	2.65 ± 0.02	3.21 ± 0.03	3.18 ± 0.03	3.18 ± 0.02	3.31 ± 0.06^+^
% predicted	98.44 ± 0.59	102.86 ± 0.54	102.47 ± 0.53	99.09 ± 0.60^&^	100.91 ± 0.43	101.01 ± 1.05
						
**Observed FVC (L)**	4.73 ± 0.04	3.39 ± 0.02	3.97 ± 0.04	3.98 ± 0.04	3.95 ± 0.03	4.14 ± 0.07^+^
% predicted	98.55 ± 0.55	103.24 ± 0.51	102.38 ± 0.50	99.77 ± 0.57^&^	101.10 ± 0.41	101.76 ± 0.97
						
**Observed FEV**_1_**/FVC**	0.80 ± 0.002	0.81 ± 0.002	0.81 ± 0.0.002	0.80 ± 0.0.002^&^	0.80 ± 0.002	0.80 ± 0.004
% predicted	99.96 ± 0.29	100.06 ± 0.25	100.39 ± 0.26	99.57 ± 0.27^&^	100.11 ± 0.20	99.44 ± 0.49
						
**Observed FEF**_25-75_**(L/s)**	3.82 ± 0.05	2.89 ± 0.03	3.36 ± 0.04	3.21 ± 0.04^&^	3.28 ± 0.03	3.37 ± 0.08
% predicted	101.28 ± 1.10	103.89 ± 1.06	105.64 ± 1.04	99.30 ± 1.13^&^	102.89 ± 0.82	101.80 ± 2.17

Note: Predicted values are adjusted for age and height.

* Comparison made between Male and Female p value < 0.05

** Comparison made between Male and Female p value < 0.10

^+ ^Comparison made between wild type and polymorphic group p value < 0.05

^&^Comparison made between never smoker and ever smoker group p value < 0.05

^# ^Separate prediction equations used for males and females.

**Figure 1:**
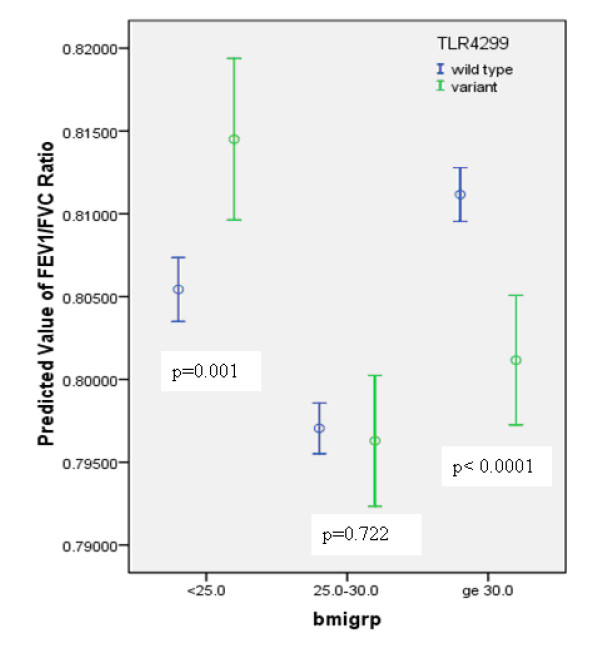
**Association between TLR4 genotype and FEV_1_/FVC ratio by BMI categories (error bars represent 95% confidence intervals around the mean)**.

**Figure 2:**
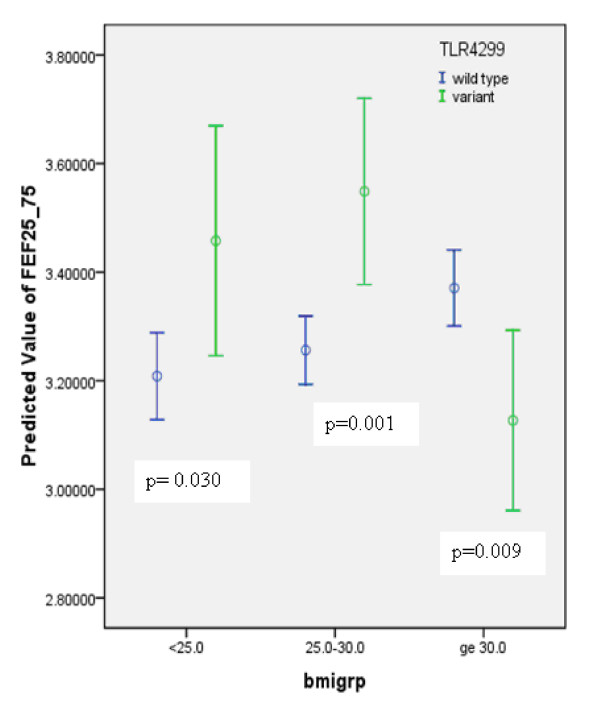
**Association between TLR4 genotype and FEF_25-75 _by BMI categories (error bars represent 95% confidence intervals around the mean)**.

Results of the univariate analysis are shown in Table 3. Variables with p-value < 0.20 were included in the multivariable model. However, TLR4 Asp299Gly was a risk factor of primary interest, so it was kept in all the four final models selected to draw conclusions (one model for each of lung function outcome variable: FEV_1_, FVC, FEV_1_/FVC ratio, FEF_25-75_). The significant predictors for FEV_1 _and FVC were: age, smoking status, and TLR4 299 genotype (see Table 4). For FEV_1_, BMI achieved borderline significance. For FEV_1_/FVC ratio and FEF_25-75_significant predictors were: age smoking status, and sex. BMI was an effect modifier for the relationship between TLR4 Asp299Gly and FEV_1_/FVC ratio and FEF_25-75_. For different categories of BMI, the relationship between (i) TLR4 Asp299Gly and FEV_1_/FVC ratio, and (iii) TLR4 Asp299Gly and FEF_25-75 _are shown in Figures 1, and 2 respectively. Due to multiple comparisons, adjustment in p-values (0.05/3 = 0.01667 was used for statistical significance) was made based on the Bonferroni correction.

**Table 3:** Univariate regression analysis (one variable at a time) for relationship between lung function and TLR4 wild-type and polymorphic groups

**Lung Function**	**Model I** **FEV_1_**	**Model II** **FVC**	**Model III** **FEV1/FVC**	**Model IV** **FEF25_75**
**Variable**	**Parameter Estimate (SE)**	**Parameter Estimate (SE)**	**Parameter Estimate (SE)**	**Parameter Estimate (SE)**

**Age, yrs**	-0.033 (0.001)***	-0.036 (0.001)***	-0.001 (0.000)***	-0.041 (0.002)***
**BMI**^‡ ^(reference is normal)				
Overweight	0.074 (0.055)	0.134 (0.067)*	-0.010 (0.004)*	0.057 (0.075)
Obese	-0.060 (0.057)	-0.106 (0.069)	0.003 (0.004)	0.096 (0.077)
**Smoking status **(reference is never smoker)				
Ever smoker	-0.032 (0.044)	0.012 (0.054)	-0.011 (0.003)**	-0.151 (0.060)*
**Sex **(reference is female)				
Male	1.031 (0.037)***	1.336 (0.044)***	-0.011 (0.003)**	0.934 (0.056)***
**TLR4299 **(reference is wild-type)				
polymorphic groups	0.134 (0.064)*	0.185 (0.078)*	-0.002 (0.004)	0.091 (0.088)
**Grain Dust **(reference is not exposed)				
Exposed	0.299 (0.045)***	0.426 (0.054)***	-0.011 (0.003)**	0.214 (0.061)**
**Household Smoking **(Reference is No)				
Yes	0.021 (0.069)	0.001 (0.083)	0.004 (0.005)	0.037 (0.093)

*** p < 0.001; ** p < 0.01; * p < 0.05

^‡^Normal: <25; Overweight: 25-30; Obese: >30

**Table 4:** Multiple regression analysis for relationship between lung function and TLR4 wild-type and polymorphic groups, adjusting for age, BMI, sex and smoking

**Lung Function**	**FEV_1_**	**FVC**	**FEV1/FVC**	**FEF25_75**
Variable	Parameter Estimate (SE)	Parameter Estimate (SE)	Parameter Estimate (SE)	Parameter Estimate (SE)

**Age, yrs**	-0.033 (0.001)***	-0.036 (0.001)***	-0.001 (0.000)***	-0.041 (0.002)***
**BMI **(reference is normal: <25)				
Overweight (BMI: 25-30)	0.019 (0.035)	0.026 (0.042)	-0.00002 (0.004)	0.065 (0.064)
Obese (BMI: >30)	-0.057 (0.035)	-0.145 (0.043)**	0.015 (0.004)***	0.232(0.065)***
**Smoking status **(reference is never smoker)				
Ever smoker	-0.074 (0.027)**	-0.056 (0.033)	-0.007 (0.003)*	-0.176 (0.047)***
**Sex **(reference is female)				
Male	1.059 (0.028)***	1.367 (0.034)***	-0.010 (0.003)**	0.959(0.048)***
**TLR4299 **(reference is wild-type)				
polymorphic groups	-0.020 (0.039)	0.003 (0.048)	0.007 (0.009)	0.062 (0.135)
**Interactions**				
**BMI and TLR4299** (reference is < 25 BMI and Wild type)				
25-30 BMI group and polymorphic group			-0.008 (0.011)	0.040 (0.172)
>30 BMI group and polymorphic group			-0.021 (0.011)^#^	-0.432 (0.175)*

*** p < 0.001; ** p < 0.01; * p < 0.05 ^# ^p < 0.10

## Discussion

In this study we investigated the association between TLR4 Asp299Gly polymorphism and the lung function in a community population. Based on the multivariable statistical analysis, this cross-sectional study showed that TLR4 Asp299Gly was a potential risk factor for reduced FEV_1_/FVC and FEF_25-75 _in obese subjects (BMI ≥ 30 kg/m^2^). Earlier studies have shown: (i) an association between obesity and reduced lung function [12-15,23,24]; (ii) metabolic syndrome and impaired lung function [16,17]; (iii) no association between TLR4 polymorphism and metabolic syndrome and diabetes [10,11]; and (iv) controversial relationship between TLR4 polymorphism and lung function [2-7,9,25]. The most important finding of this report is that BMI appears to be an effect modifier in the relationship of TLR4 polymorphism and lung function. Based on the multivariable analysis: (i) the variant group had a significantly higher mean predicted FEV_1_/FVC ratio in the BMI category < 25 compared to the wild type group; (ii) no difference between wild type and variant groups in the BMI category 25 - <30; and (iii) the polymorphic group had lower mean predicted FEV_1_/FVC ratio compared to the wild type group in the BMI category ≥ 30. Conversely, the wild type group had significantly lower mean value of predicted FEF_25-75 _in BMI category 25 - <30 and significantly higher mean value of FEF_25-75 _in the BMI category ≥ 30 compared to the polymorphic group.

In a recent study by Pistelli et al. [23] it was reported that the "becoming obese" and "always obese" categories had a significantly greater decline in lung function than "never obese" group. In the "always obese" group, this was true for vital capacity but not for FEV_1_. In our report, we did not observe any significant interaction between BMI and TLR4 gene for FVC and FEV_1_. However the relationship between obesity (in terms of BMI) and lung function indices FEV_1_/FVC ratio and FEF_25-75 _was different between wild type and polymorphic groups. In another recent study, Rohde et al. [25] reported that the frequency of TLR4 Asp299Gly polymorphism is decreased in COPD patients. Cross-sectional analysis of our database did not show any association between TLR4 polymorphism and COPD. Reasons for the lack of association between TLR4 polymorphism and COPD may be either a small sample size or a low frequency of TLR4 Asp299Gly polymorphism in the study population. To examine completely the role of TLR4 Asp299Gly polymorphism, large populations or longitudinal studies are required to give adequate statistical power to test the hypothesis of association between this TLR4 polymorphism and COPD.

Santos et al suggest that it is unlikely that TLR4 Asp299Gly polymorphisms have a major influence on adiposity, bone mineral density or osteoporosis status in Chilean elderly women [10]. A similar finding of no association between TLR4 Asp299Gly and metabolic syndrome or diabetes was observed by Illig et al. [11] Research has shown that obesity affects the mechanics and physiology of the respiratory system and people with higher BMI [18,23] and waist circumference [17] are more likely to have lower values for FEV_1_, FVC and higher values for FEV_1_/FVC ratio. A study conducted in Taiwan reported that obesity and metabolic syndrome were associated with impaired lung function in an adult population [16]. It was suggested by the authors that obesity and insulin resistance may be the common pathways underlying lung function impairment and metabolic syndrome [16]. Shen et al. reported that waist circumference correlates with metabolic syndrome and other health risk indicators, followed by BMI [17]. The authors also reported that while percent fat is a useful indicator of overall adiposity, health risks are best represented by the simply measured waist circumference [17]. In a recent study, Ochs-Balcom et al. [24] reported that abdominal adiposity is a better predictor of pulmonary function than weight or BMI. The etiology of metabolic syndrome is complex, which is determined by the interplay of genetic and environmental factors [26]. Yamada et al identified a genetic polymorphism that conferred susceptibility to metabolic syndrome [26]. Illig et al. reported that there is no impact of TLR4 polymorphism on major features of metabolic syndrome [11]. According to the National Cholesterol Education Program's Adult Treatment Panel III report (ATP III) metabolic syndrome can be identified clinically based on the five components: abdominal obesity, given as waist circumference (for men > 102 cm and for women > 88 cm); triglycerides (≥ 150 mg/dL); high-density lipoprotein choelstrol (< 40 mg/dL); blood pressure (≥ 130/≥ 85 mm Hg) and fasting glucose (≥ 110 mg/dL). Abdominal obesity is most strongly associated with the metabolic syndrome [27]. In our study, information on all the five components of metabolic syndrome was not available to us, hence, when we conducted the statistical analysis by replacing BMI with waist circumference in multivariable models, and similar results were obtained.

In our study TLR4 Asp299Gly frequency was 15.8% for males and 11.9% for females, so it was of interest to explore the three-way interaction between sex, TLR4 and BMI. A 3-way crosstab analysis showed that there may be a potential three-way interaction between sex, TLR4 Asp299Gly polymorphism and BMI. There was no significant difference in the mean values of FEV_1 _and FVC for wild and variant groups when stratified by sex. Mean values for predicted FEV_1_/FVC ratio were (i) significantly higher in variant group (males: 80.9 ± 0.02, females: 81.8 ± 0.02) compared to the wild type (males: 79.9 ± 0.02, females: 80.8 ± 0.02) in BMI category <25 kg/m^2 ^and (ii) significantly lower for the variant group (males: 79.5 ± 0.02, females: 80.6 ± 0.02)) compared to the wild type group (males: 80.6 ± 0.02, females: 81.6 ± 0.02) in BMI category ≥ 30 kg/m^2^. Mean values for predicted FEF_25-75 _were (i) significantly higher for the variant group (males: 3.93 ± 0.69, females: 3.71 ± 0.63) compared to the wild type group for BMI category 25 - < 30 kg/m^2 ^; and (ii) significantly lower in the variant group (3.62 ± 0.68) compared to the wild type group (3.89 ± 0.63) in BMI category ≥ 30 kg/m^2^. The association between TLR4 Asp299Gly polymorphism and FEF_25-75 _was not modified by BMI for females. However, this three-way interaction was not significant in the multivariable regression model, possibly due to a relatively low prevalence of TLR4 Asp299Gly in the study population.

This study had some methodological strengths and as well as weaknesses. The pulmonary function tests were administered by trained nurses using techniques which conformed to ATS standards. The pulmonary function tests were reviewed by a respiratory physician. A weakness of this study was that the number of the subjects was too small to conduct comparisons among three groups (Asp299Asp, Asp299Gly and Gly299Gly). For TLR4 299, 86.4%, were Asp299Asp, 13.4% were Asp299Gly, and 0.2% were Gly299Gly [Rennie DC, Chen Y, Pahwa P, Burch LH, Bharadwaj L, Grover VK, Schwartz DA, Dosman JA: Gender-related associations between toll-like 4 receptor 299 polymorphism and respiratory outcomes, submitted]. We therefore combined the last two groups. The allele frequencies of single nucleotide polymorphisms, such as Asp299Gly, vary between different ethnic populations. The TLR-4 Asp299Gly polymorphism has different minor allele frequencies in, for example, 0% in Chinese, 6.5% in American and 9.8% in West African populations [28]. 93% of our study subjects were Caucasians; 0.4% were aboriginal; 1.4% had reported other ethnicity, and for 5.3% subjects ethnicity was missing. We were unable to define the presence/absence of metabolic syndrome due to lack of information on all the five components of metabolic syndrome and hence conducted our analysis using BMI (and repeated statistical analysis using waist circumference [results not shown]).

Overall, our study was adequately powered to detect clinically meaningful differences. To our knowledge this is the first study which showed that BMI is an effect modifier in the relation of TLR4 Asp299Gly polymorphism and lung function (FEV_1_/FVC ratio and FEF_25-75_). More studies are needed to accumulate the evidence of this effect modification and to assess the credibility [29] of this association. It is essential to understand and investigate the interplay between lung function, TLR4 Asp299Gly polymorphism and metabolic syndrome in humans. TLR4 itself is not associated with lung function but has opposing effects at extremes of BMI. The mechanism is unknown to explain these opposing effects. Further studies should investigate the role of TLR4 Asp299Gly and additional polymorphisms of TLR4 to understand the potential effects of innate immunity on lung function in relation to metabolic syndrome. It will be important to investigate whether or not TLR4 Asp*299*Gly linked to another genetic modifier of the metabolic syndrome. The association of TLR4 Asp299Gly variant status and lung function should be replicated in more than one population in order to establish the importance of this finding.

## Conclusion

BMI may modify the associations of TLR4 Asp299Gly polymorphism with FEV_1_/FVC ratio and FEF_25-75_. The association of TLR4 Asp299Gly variant status and lung function should be analyzed in more than one population in order to establish the importance of this finding.

## Abbreviations

TLR: Toll-like receptor; FEV_1_: Forced expiratory volume in the first second; FVC: Forced vital capacity; FEF: forced expiratory flow rate; BMI: Body mass index, DNA: Deoxyribonucleic acid; COPD: Chronic Obstructive Pulmonary Disease; MGB: Minor groove binding; NFQ: Non-fluorescent quencher; (SE): Standard Error.

## Competing interests

The authors declare that they have no competing interests.

## Authors' contributions

PP designed and coordinated the study as well as data collected and prepared the manuscript. CPK analyzed data and prepared the manuscript. DCR participated in the design of the study and data collection and prepared the manuscript. YC participated in study design, coordination, and data collection. DAS involved with genetic analysis. JAD participated in study design, coordination, data collection and manuscript preparation. All authors read and approved the final manuscript.

## Pre-publication history

The pre-publication history for this paper can be accessed here:


